# A Functional Subnetwork Approach to Designing Synthetic Nervous Systems That Control Legged Robot Locomotion

**DOI:** 10.3389/fnbot.2017.00037

**Published:** 2017-08-09

**Authors:** Nicholas S. Szczecinski, Alexander J. Hunt, Roger D. Quinn

**Affiliations:** ^1^Biologically Inspired Robotics Laboratory, Department of Mechanical and Aerospace Engineering, Case Western Reserve University, Cleveland, OH, United States; ^2^Department of Mechanical and Materials Engineering, Portland State University, Portland, OR, United States

**Keywords:** synthetic nervous system, design tools, functional subnetworks, leaky integrator, arithmetic, differentiator, memory

## Abstract

A dynamical model of an animal’s nervous system, or synthetic nervous system (SNS), is a potentially transformational control method. Due to increasingly detailed data on the connectivity and dynamics of both mammalian and insect nervous systems, controlling a legged robot with an SNS is largely a problem of parameter tuning. Our approach to this problem is to design functional subnetworks that perform specific operations, and then assemble them into larger models of the nervous system. In this paper, we present networks that perform addition, subtraction, multiplication, division, differentiation, and integration of incoming signals. Parameters are set within each subnetwork to produce the desired output by utilizing the operating range of neural activity, *R*, the gain of the operation, *k*, and bounds based on biological values. The assembly of large networks from functional subnetworks underpins our recent results with MantisBot.

## 1 Introduction

The development of robotic control that can closely match the dexterity and adaptability found in the animal kingdom has so far remained elusive. This is because the control of locomotion is a complex process controlled by dynamic systems which are not fully understood. However, recent advances in neural imaging and recording has lead to an increase in the abundance and detail of our knowledge of how an animal’s nervous system controls its body within the context of its environment (for a recent review, see Buschmann et al. (2015)).

These advances have lead to an explosion of bio-inspired robotic systems in recent years (for a review, see Ijspeert (2014)). These models can be broadly categorized into a range of template and anchor models. In a template model, biological principles are abstracted, such using as a spring-loaded inverted pendulum (SLIP) model to investigate bipedal locomotion (Blickhan, 1989) or using Whegs to investigate insect locomotion (Allen et al., 2003; Schroer et al., 2004). These models seek to explain how specific characteristics of animal locomotion lead to desired behaviors, or they exploit certain principles of animal locomotion for more agile robotic systems. Anchor models, in contrast, seek to directly mimic particular mechanical or control mechanisms from animals, in order to understand how they function. Robots such as Pleurobot (Karakasiliotis et al., 2016), Puppy (Hunt et al., 2017), MantisBot (Szczecinski and Quinn, 2017; Szczecinski et al., 2017a), and others are relevant anchor models, because they seek to use highly articulated robots with central pattern generator controllers to understand how specific animals are capable of providing adaptable locomotion with their unique morphology and physical constraints.

The template versus anchor model distinction is not limited to physical models; it can also be applied to control systems. The majority of robotic controllers so far have been template models, either mathematical abstractions of neural systems, or black box artificial neural networks. This is because effective tools for setting parameters in more realistic, dynamic neural models to produce reliable behavior in a robotic system do not yet exist. In spite of growing knowledge about the neural connectivity that underlies locomotion control, detailed data for tuning these systems (neural time constants, ion channel conductivities, synaptic conductivities, etc.) remain largely unavailable, requiring the modeler or engineer to tune these parameter values. However, this is an inherently difficult task because there are many parameters to be tuned in a model, and likely many different parameter combinations that lead to indistinguishable performance (Prinz et al., 2004; Marder and Taylor, 2011). Thus, the emphasis in choosing parameter values should not be on selecting the singular “correct” values, but rather sufficiently “effective” values. In this work, we tune parameter values in functional subnetworks for addition, subtraction, multiplication, division, differentiation, and integration of incoming signals and use analytical techniques to identify *constraints* on the parameter values that must be met for the intended calculations to occur. Larger networks can then be assembled from these subnetworks with no additional tuning (Szczecinski and Quinn, 2017; Szczecinski et al., 2017a). In this manuscript, “tuning” refers to selecting the static parameter values for a network; “learning” refers to changing the parameter values while the model performs a task, either in simulation or in a robot, based on its performance; and “adapting” refers to a model qualitatively changing its behavior (e.g., walking more slowly), either with or without “learning.”

Neuromechanical models may be tuned in a supervised or unsupervised way. A supervised tuning method adjusts the parameters of the model until the model replicates animal data. This includes tuning the model by hand (Daun-Gruhn and Tóth, 2010; Szczecinski et al., 2014; Markin et al., 2016), which is a time-consuming and imprecise process. Such imprecision may be acceptable in simulation studies, but provides many difficulties for robots that must interact with real environments. Techniques do exist for tuning controllers based on animal locomotion data (Schilling et al., 2013; Hunt et al., 2015b, 2017; Karakasiliotis et al., 2016). However, collecting kinematic and dynamic data from animals is time-consuming and expensive, and once collected, must be further processed to scale the dynamics of the animal to the robot (Karakasiliotis et al., 2016; Hunt et al., 2017). In addition, using cross-individual average values for tuning dynamical neural models may fail in many cases, because the average may not represent any one individual (Golowasch et al., 2002; Marder and Taylor, 2011). Large amounts of animal data may be used to tune a control network of abstracted artificial neural networks (Schilling et al., 2013). Methods like back-propagation can be used to adjust synaptic weights in the network until it captures the animal data arbitrarily closely, if it has enough connections (Trappenberg, 2009). However, because the control network is abstracted, so are the biological insights gained from the model.

Unsupervised tuning methods, in contrast, tune the model based on how well the model accomplishes a task, such as navigating toward a goal, without comparison to animal data. These methods frequently use genetic algorithms (GAs) (Beer and Gallagher, 1992; Haferlach et al., 2007; Agmon and Beer, 2013; Izquierdo and Beer, 2013) or reservoir computing (RC) (Dasgupta et al., 2015) to test many different networks and parameter values, based on a simulated agent’s performance. GAs can be effective at finding networks that perform specific operations, such as oscillating (Beer and Gallagher, 1992), navigating (Haferlach et al., 2007), or switching between foraging tasks (Agmon and Beer, 2013). However, this approach has some drawbacks. Specifically, the evolution process is slow, requiring the simulation of hundreds or thousands of parameter combinations (Agmon and Beer, 2013), which may take days without great computing power. The speed and likelihood of success can be increased by embedding functional subnetworks in the network (Pasemann et al., 2001; Haferlach et al., 2007), which may be identified by brute-force (Prinz et al., 2003), dynamical systems analysis (Hunt et al., 2017), or constraints on network connectivity and parameter values (Haferlach et al., 2007). In this paper, we analytically derive parameter constraints to eliminate the need for GAs altogether, and guarantee network performance.

RC methods simulate large “reservoirs” of randomly connected dynamical neuron models, and then use optimization methods to map reservoir activity to learned useful values. While this method can produce capable robotic controllers (Dasgupta et al., 2015), the final system is likely more complicated than is ultimately necessary, increasing its computational cost to implement. In addition, the final system is a black box, which does not provide any insights about nervous system function. The methods in this paper enable the direct assembly and tuning of dynamical networks without the need of large reservoirs of neurons.

This work analytically derives constraints that govern the behavior of synthetic nervous systems (SNSs) built from dynamical neural networks. These constraints were derived as a result of our previous network design work (Szczecinski et al., 2017b) and have enabled the rapid assembly and testing of our recent robot control networks (Szczecinski and Quinn, 2017; Szczecinski et al., 2017a). An SNS designer can apply these constraints to find parameter values needed for a functional network. Section 2 presents the neural and synaptic models and explains how the neural system encodes mechanical inputs and outputs. Section 3 derives two basic synapse types, “signal transmission” and “signal modulation,” and uses them to derive constraints on synaptic parameters in networks performing addition, subtraction, multiplication, and division of two incoming signals. Section 4 derives constraints on neural and synaptic parameters in networks that differentiate and integrate incoming signals as a function of time. Results showing that the networks perform as intended are provided throughout the manuscript, and Tables 1 and 2 summarize the design constraints. Finally, Sec. 6 explores how these techniques may be used to tune robot controllers and neuromechanical models of animals, and how they may be improved in the future.

**Table 1:** This table assumed that the designer has already selected a value of *R* for the subnetwork.

Operation	Componentpathways	Constraint equations	Free parameters
Addition	Syn. 1,	$g_{s\text{,1}}=\frac{k_{\mathrm{syn}\text{,1}}\cdot R}{\Delta E_{s\text{,1}}-k_{\mathrm{syn}\text{,1}}\cdot R}$	*k*_*syn*,1_
	transmission	Δ*E*_*s*,1_ − *k*_*syn*,1_⋅*R* > 0	Δ*E*_*s*,1_, maximize
	Syn. 2,	$g_{s\text{,2}}=\frac{k_{\mathrm{syn}\text{,2}}\cdot R}{\Delta E_{s\text{,2}}-k_{\mathrm{syn}\text{,2}}\cdot R}$	*k*_*syn*,2_
	transmission	Δ*E*_*s*,2_ − *k*_*syn*,2_⋅*R* > 0	Δ*E*_*s*,2_, maximize

Subtraction	Syn. 1,	$g_{s\text{,1}}=\frac{k_{\mathrm{syn}}\cdot R}{\Delta E_{s\text{,1}}-k_{\mathrm{syn}}\cdot R}$	*k_syn_*
	transmission	Δ*E*_*s*,1_ − *k_syn_*⋅*R* > 0	Δ*E*_*s*,1_, maximize
	Syn. 2,	$g_{s\text{,2}}=\frac{\Delta E_{s\text{,1}}}{\Delta E_{s\text{,2}}}\cdot \frac{-k_{\mathrm{syn}}\cdot R}{\Delta E_{s\text{,1}}-k_{\mathrm{syn}}\cdot R}$	Δ*E*_*s*,2_, minimize
	transmission		

Division	Syn. 1,	$g_{s\text{,1}}=\frac{k_{\mathrm{syn}}\cdot R}{\Delta E_{s\text{,1}}-k_{\mathrm{syn}}\cdot R}$	*c_syn_*
	transmission	*k_syn_* = 1	Δ*E*_*s*,1_, maximize
		Δ*E*_*s*,1_ − *k_syn_*⋅*R* > 0	
	Syn. 2,	$g_{s\text{,2}}=\frac{c_{\mathrm{syn}}\cdot R-R}{\Delta E_{s\text{,2}}-c_{\mathrm{syn}}\cdot R}$	
	modulation	Δ*E*_*s*,2_ = 0	
		0 < *c_syn_* < 1	

Multiplication	Syn. 1,	$g_{s\text{,1}}=\frac{k_{\mathrm{syn}}\cdot R}{\Delta E_{s\text{,1}}-k_{\mathrm{syn}}\cdot R}$	
	transmission	*k_syn_* = 1	
		Δ*E*_*s*,1_ − *k_syn_*⋅*R* > 0	Δ*E*_*s*,1_, maximize
	Syn. 2,	$g_{s\text{,2}}=\frac{-R}{\Delta E_{s\text{,2}}}$	
	modulation	Δ*E*_*s*,2_ < 0	Δ*E*_*s*,2_, maximize
	Syn. 3,	*g*_*s*,3_ = *g*_*s*,2_	
	modulation	Δ*E*_*s*,3_ = Δ*E*_*s*,2_	

*In this table, “minimize” refers to making a value as negative as possible and “maximize” refers to making a value as positive as possible*.

**Table 2:** This table assumed that the designer has already selected a value of *R* for the subnetwork.

Operation	Components	Constraints and useful relations	Free parameters
Differentiation	Neuron 1	*C*_*m*,1_ < *C*_*m*,2_	τ*_d_*
	Neuron 2	*C*_*m*,2_ = τ*_d_*	*k_d_*
		*C*_*m*,1_ = *C*_*m*,2_ − *k_d_*	
	Syn. 1,	*k_syn_* = 1/*k_d_*	Δ*E*_*s*,1_, maximize
	transmission	$g_{s\text{,1}}=\frac{k_{\mathrm{syn}}\cdot R}{\Delta E_{s\text{,1}}-k_{\mathrm{syn}}\cdot R}$	
		Δ*E*_*s*,1_ − *k_syn_*⋅*R* > 0	
	Syn. 2,	$g_{s\text{,2}}=\frac{\Delta E_{s\text{,1}}}{\Delta E_{s\text{,2}}}\cdot \frac{-k_{\mathrm{syn}}\cdot R}{\Delta E_{s\text{,1}}-k_{\mathrm{syn}}\cdot R}$	Δ*E*_*s*,2_, minimize
	transmission		

Integration	Neuron 1	*I*_*app*,1_ = *R*	*k*_*i*,mean_
		$C_{m\text{,1}}=\frac{\text{1}}{\text{2}\cdot k_{i,\text{mean}}}$	
	Neuron 2	*I*_*app*,2_ = *R*	
		*C*_*m*,1_ = *C*_*m*,2_	
	Syn. 1,	$\Delta E_{s\text{,1}}=\frac{-R}{g_{s\text{,1}}}$	*k*_*i*,range_
	transmission	$g_{s\text{,1}}=\frac{\text{2}\cdot C_{m\text{,1}}}{\text{1}∕k_{i\text{,range}}-C_{m\text{,1}}}$	
	Syn. 2,	*g*_*s*,2_ = *g*_*s*,1_	
	transmission	Δ*E*_*s*,2_ = Δ*E*_*s*,1_	

*In this table, “minimize” refers to making a value as negative as possible and “maximize” refers to making a value as positive as possible*.

## 2 Methods: Models and Approach

We model neurons as non-spiking Hodgkin–Huxley compartments (Cofer et al., 2010), the same basic model as used in continuous-time recurrent neural networks (Haferlach et al., 2007; Agmon and Beer, 2013). The leaky integrator dynamics capture the most basic behavior of neurons and allow more complex behaviors to be added with additional ion channels, if desired. This work is not concerned with the specifics on how action potentials are generated and have left out Hodgkin–Huxley sodium and potassium currents. The membrane voltage, *V*, may be seen as a proxy for the spiking frequency of a spiking neuron. *V* varies according to the differential equation

(1) $$C_{m}\frac{\mathrm{dV}}{\mathrm{dt}}=I_{\mathrm{leak}}+I_{\mathrm{syn}}+I_{\mathrm{app}}$$

where

(2) $$I_{\mathrm{leak}}=G_{m}\cdot \left(E_{r}-V\right),$$

(3) $$I_{\mathrm{syn}}=\sum_{i=1}^{n}G_{s,i}\cdot \left(E_{s,i}-V\right),$$

and *I_app_* is an optional external stimulus. Equations (2) and (3) define the leak and synaptic currents, respectively. Both follow the same basic form of a conductance *G* multiplied by the difference between the current membrane voltage, *V*, and a constant reference voltage (i.e., reversal potential), *E*. *E_r_* is the resting potential of the neuron, and *C_m_* and *G_m_* are the capacitance and conductance of the cell membrane, respectively. Unless otherwise noted, all units in this paper are scaled to nA for current, mV for potentials, nF for capacitances, and μS for conductances.

Neurons communicate via synapses. The conductance, *G_s,i_* in equation (3), is a threshold linear function of the *i*th incoming (i.e., presynaptic) neuron’s voltage. Synapses communicate via piecewise-linear functions described as

(4) $$G_{s,i}=\left\{\begin{array}{ll} 0,\; & \text{if}\;V_{\mathrm{pre}}<E_{\mathrm{lo}},\\ g_{s,i}\cdot \frac{V_{\mathrm{pre}}-E_{\mathrm{lo}}}{E_{\mathrm{hi}}-E_{\mathrm{lo}}},\; & \text{if}\;E_{\mathrm{lo}}<V_{\mathrm{pre}}<E_{\mathrm{hi}},\\ g_{s,i},\; & \text{if}\;V_{\mathrm{pre}}>E_{\mathrm{hi}}. \end{array}\right.$$

**Figure 1:**
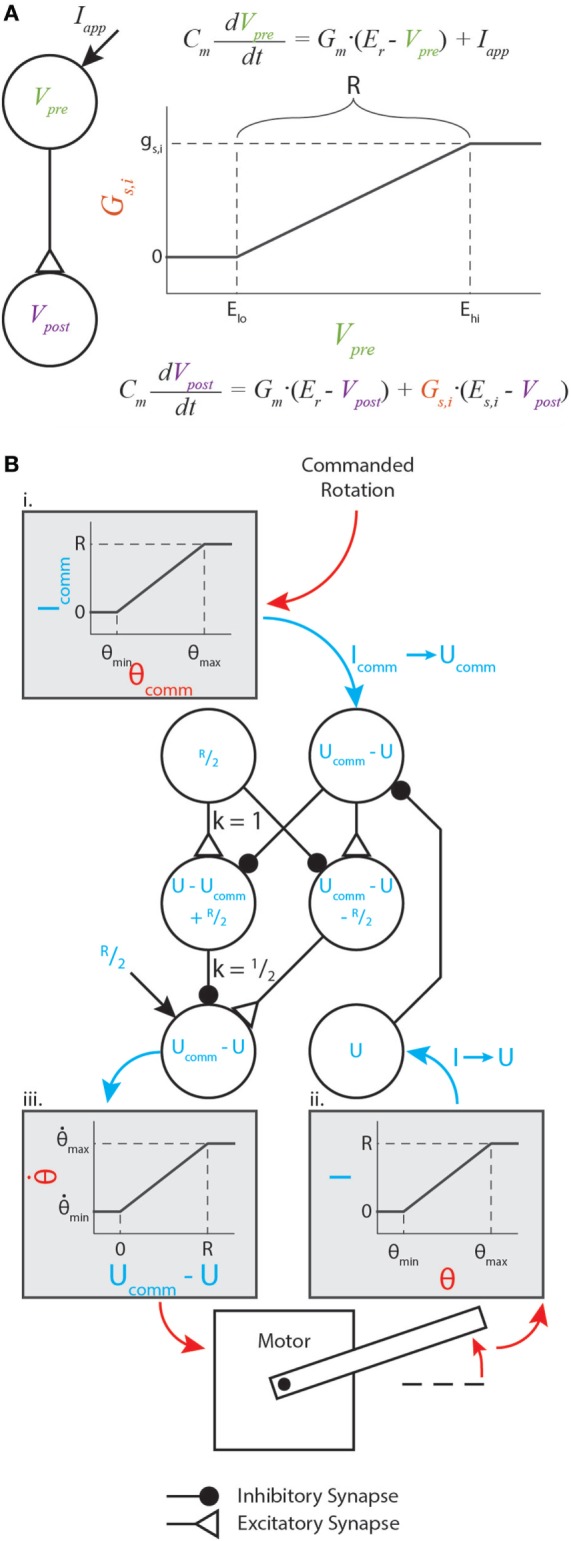
Graphical representation of synaptic dynamics and mapping between mechanical and neural values. **(A)** Graphical representation of how synapses couple neural dynamics. Note that *R* is marked on the plot. **(B)** Enhanced version of the motor control network from Szczecinski et al. (2017b), showing how *R* relates mechanical and neural values. Mechanical values are drawn in red, and neural values are drawn in blue. Gray shaded boxes map from mechanical to neural values (i. and ii.), or from neural to mechanical values (iii.).

The parameters *g_s,i_, E_lo_*, and *E_hi_* are constants representing the synapse’s maximum conductance, its lower threshold, and its upper threshold, respectively. The relationship between the presynaptic neuron voltage, synaptic conductance, and postsynaptic neuron voltage is illustrated in Figure 1A.

We prefer this piecewise-linear representation better than a sigmoidal function for several reasons. First, the thresholds ensure that for low activations, synapses conduct exactly 0 current. This could represent a reduced model of a spiking neuron, which transmits no information while it is not spiking. Second, equation (4) contains no transcendental terms, facilitating analytical manipulation of the equations. A discontinuous system does complicate traditional gradient-based optimization methods, but this structure can be exploited to make these methods unnecessary. In the following sections, we show how networks of three or four neurons with synapses between them can be constructed and analytically tuned to perform mathematical operations on the input signals, such as addition or differentiating with respect to time.

Instead of analyzing *V* when designing these networks, we shift the neural activity to simplify analysis. For each neuron, we substitute *U* = *V* − *E_r_*, the activation level above the resting voltage. A typical value is *E_r_* = −60 mV, but using *U* for analysis rather than *V* lets us apply the same analysis no matter what *E_r_* is. We also set *G_m_* = 1 μS, which is a typical value (Daun-Gruhn et al., 2009; Daun-Gruhn, 2010).

For the synapses, we set *E_lo_* = *E_r_* of the presynaptic neuron, and introduce a new parameter *R* = *E_hi_* − *E_lo_*. Thus, a synapse’s conductivity rises as the presynaptic neuron’s voltage rises above its resting potential, and exhibits an “operating range” of *R* mV. The constraints we apply ensure that *U_pre_* ∈ [0, *R*], meaning that the synapse is always active, but never saturates. Thus, we can replace *G_s_* with the second line of equation (4). Applying the substitutions described so far,

(5) $$G_{s}=g_{s}\cdot \frac{V_{\mathrm{pre}}-E_{\mathrm{lo}}}{E_{\mathrm{hi}}-E_{\mathrm{lo}}}=g_{s}\cdot \frac{U_{\mathrm{pre}}}{R}=\frac{g_{s}}{R}\cdot U_{\mathrm{pre}}.$$

For each synapse, we also introduce the parameter Δ*E_s,i_* = *E_s,i_* − *E_r,post_*, where *E_r,post_* is the resting potential of the postsynaptic, or receiving neuron.

Making all of these substitutions in equations (1)–(3) gives the response

(6) $$C_{m}\frac{\mathrm{dU}}{\mathrm{dt}}=-U+\sum_{i=1}^{n}\frac{g_{s,i}}{R}\cdot U_{\mathrm{pre},i}\cdot \left(\Delta E_{s,i}-U\right)+I_{\mathrm{app}}.$$

When *U* = *R*, the neuron is fully active, and when *U* = 0, the neuron is inactive. We can use this knowledge to categorize synapses as excitatory or inhibitory, depending on the sign of Δ*E_s,i_*. If Δ*E_s,i_* ≥ *R*, then the *i*th synapse will always transmit positive current, no matter the instantaneous value of *U*. Thus, this synapse will cause *U* to increase and is, therefore, excitatory. Similarly, if Δ*E_s,i_* ≤ 0, then the *i*th synapse will always transmit negative current, no matter the instantaneous value of *U*. Thus, this synapse will cause *U* to decrease and is, therefore, inhibitory.

### 2.1 Mapping between Neural and Mechanical Values

The nervous system encodes physical quantities as neural activity. In insects, the firing rate of sensory neurons encode the stretch of chordotonal organs (Field and Matheson, 1998) and the strain of campaniform sensilla (Zill et al., 2004), among other physical quantities. Typical robot controllers perform operations on these signals to provide meaningful information for control actions. These operations may include the subtraction of measured and reference values, differentiation or integration of error values, or gain adjustments. Neural systems perform these same operations, but in a transformed space. The exact transformation that nervous systems use is not known, but for reliable behavior, it is necessary that sensory information is mapped to neural activity in apredictable way. Thus, we map any sensory input, *θ*, to an applied current

(7) $$I_{\mathrm{app}}=R\cdot \frac{\theta -\theta_{\min}}{\theta_{\max}-\theta_{\min}},$$

where *R* is the “operating range” specified in the previous section. Figure 1B illustrates such a transformation within a diagram of a neural feedback loop that controls the position of a motor. The purpose of this paper is not to analyze how this particular network functions; for a detailed analysis of this network and its function, see Szczecinski et al. (2017b). Instead, the purpose is to show how *R* and other functional values (time constants, gains, etc.) may be used to constrain neural and synaptic parameter values.

Figure 1B (i,ii) graphically illustrate the mapping in equation (7), and Figure 1B (iii) graphically illustrates the inverse relationship (i.e., neural value to mechanical value). If a sensory neuron has only this applied current and leak current, equation (6) shows that

(8) $$C_{m}\frac{\mathrm{dU}}{\mathrm{dt}}+U=R\cdot \frac{\theta -\theta_{\min}}{\theta_{\max}-\theta_{\min}}.$$

This means that the sensory neuron acts as a low-pass filter with time constant τ = *C_m_*. It is trivial to show that, when the neuron is at equilibrium (i.e., *dU*/*dt* = 0),

(9) $$U^{*}=R\cdot \frac{\theta -\theta_{\min}}{\theta_{\max}-\theta_{\min}},$$

where the superscript “∗” specifies the equilibrium value. (Throughout this manuscript, the equilibrium activation of neuron *U* will be referred to as U*, and the neuron itself will be referred to as *U*.) Equation (9) means that the neuron’s activation above its rest potential encodes the sensory signal. In addition to perceiving sensory information, commands must be issued in the same transformation. Thus, we map the commanded sensory quantity, *θ*_comm_, to the commanded neural activation, *U*_comm_, with the inverse function of equation (9),

(10) $$\theta_{\text{comm}}=\theta_{\min}+\frac{U_{\text{comm}}}{R}\cdot \left(\theta_{\max}-\theta_{\min}\right).$$

In this way, the nervous system may specify an intended motion, such as the rotation of a joint, encoded in neural activity. In our synthetic nervous systems, *R* specifies how mechanical quantities and neural activation are related. Thus, the tuning of every functional subnetwork described in this work relies on *R*, which the designer specifies before tuning the rest of the network. Two other parameters are critical for tuning these subnetworks: the amplification of synaptic transmission, *k_syn_* (discussed in Sec. 3.1), and the synaptic reversal potential, Δ*E_s_*. From these values, biological parameters such as synaptic conductance and neural tonic drive can be directly calculated. This makes network design intuitive, enabling the designer to select biological parameter values based on functional ones.

## 3 Methods: Arithmetic Subnetworks

This section describes how to use typical engineering quantities to design neural and synaptic pathways. We can understand how these pathways work by manipulating their equilibria, something that naive optimization does not leverage. The steady-state activation U* is calculated by solving for *U* when *dU*/*dt* = 0

(11) $$0=-U^{*}+\sum_{i=1}^{n}\frac{g_{s,i}}{R}\cdot U_{\mathrm{pre},i}\cdot \left(\Delta E_{s,i}-U^{*}\right)+I_{\mathrm{app}}.$$

Moving all U* terms to the left hand side

(12) $$U^{*}\cdot \left(1+\sum_{i=1}^{n}\frac{g_{s,i}}{R}\cdot U_{\mathrm{pre},i}\right)=\sum_{i=1}^{n}\frac{g_{s,i}}{R}\cdot U_{\mathrm{pre},i}\cdot \Delta E_{s,i}+I_{\mathrm{app}}.$$

Solving for U*,

(13) $$U^{*}=\frac{\sum_{i=1}^{n}\frac{g_{s,i}}{R}\cdot U_{\mathrm{pre},i}\cdot \Delta E_{s,i}+I_{\mathrm{app}}}{1+\sum_{i=1}^{n}\frac{g_{s,i}}{R}\cdot U_{\mathrm{pre},i}}.$$

This solution is the basis for the remainder of Sec. 3.

### 3.1 Signal Transmission Pathways

The goal of a signal transmission pathway is to cause the postsynaptic neuron’s voltage to be some ratio of the presynaptic neuron’s voltage. We call this ratio *k_syn_*. The *U_pre,i_* terms in the denominator of the right hand side of equation (13) mean that *k_syn_* changes as *U_pre_* changes, so we approximate *k_syn_* as *U_post_*/*U_pre_* when the presynaptic neuron is fully activated (i.e., *U_pre_* = *R*). The steady-state response of a neuron with a single synaptic input and no applied current can be written based on equation (13), as below:

(14) $$U_{\mathrm{post}}^{*}=\frac{\frac{g_{s}}{R}\cdot U_{\mathrm{pre}}\cdot \Delta E_{s}}{1+\frac{g_{s}}{R}\cdot U_{\mathrm{pre}}}.$$

To find *k_syn_* for this synapse, we first divide both sides of equation (14) by *U_pre_*,

(15) $$\frac{U_{\mathrm{post}}^{*}}{U_{\mathrm{pre}}}=\frac{\frac{g_{s}}{R}\cdot U_{\mathrm{pre}}\cdot \Delta E_{s}}{U_{\mathrm{pre}}\cdot \left(1+\frac{g_{s}}{R}\cdot U_{\mathrm{pre}}\right)}.$$

Next, we want to find *k_syn_*, which can be calculated for any value of *U_pre_*. To simplify analysis and improve the clarity of this derivation, we set find *k_syn_* when *U_pre_* = *R*. Then, we show how to set parameter values to keep *k_syn_* nearly constant, even as *U_pre_* changes. Making this substitution,

(16) $$\frac{U_{\mathrm{post}}^{*}}{R}=k_{\mathrm{syn}}=\frac{\frac{g_{s}}{R}\cdot R\cdot \Delta E_{s}}{R\cdot \left(1+\frac{g_{s}}{R}\cdot R\right)}.$$

Finally, reducing *R*/*R* terms reveals

(17) $$k_{\mathrm{syn}}=\frac{g_{s}\cdot \Delta E_{s}}{R\cdot \left(1+g_{s}\right)}.$$

Rearranging to solve for *g_s_*,

(18) $$g_{s}=\frac{k_{\mathrm{syn}}\cdot R}{\Delta E_{s}-k_{\mathrm{syn}}\cdot R}.$$

Because *g_s_* must be positive, and the numerator of equation (18) is always positive, equation (18) is also subject to the constraint

(19) $$\Delta E_{s}>k_{\mathrm{syn}}\cdot R.$$

Equation (18) will be used to tune addition, subtraction, multiplication, and division networks (Secs. 3.3 through 3.6).

### 3.2 Signal Modulation Pathways

We may also use synapses to modulate a neuron’s sensitivity to other inputs. Based on equation (13), the steady-state response of a neuron with only an applied current *I_app_* is simply

(20) $$U_{\mathrm{post}}^{*}=I_{\mathrm{app}},$$

if we set *G_m_* = 1. For example, this is the case for a sensory neuron that receives applied current proportional to a sensor’s state, such as a joint angle (Figure 1B), muscle stretch, or touch sensor. However, the nervous system may need to actively increase or reduce the sensitivity of the sensory neuron depending on context. Hyperpolarizing or depolarizing the neuron, however, would cause sensory information to be truncated (i.e., *V_pre_* < *E_lo_*). We can change the sensitivity of this neuron without losing sensory information by adding a synaptic input to the response from equation (20):

(21) $$U_{\mathrm{post}}^{*}=\frac{\frac{g_{s}}{R}\cdot U_{\mathrm{pre}}\cdot \Delta E_{s}+I_{\mathrm{app}}}{1+\frac{g_{s}}{R}\cdot U_{\mathrm{pre}}}.$$

To quantify how *U_pre_* modulates $U_{\mathrm{post}}^{*}$ for a given *I_app_*, we introduce the parameter *c_syn_*, which quantifies this degree of modulation. We define *c_syn_* as $U_{\mathrm{post}}^{*}∕U_{\mathrm{pre}}$, the same as *k_syn_*, but with the understanding that *U_pre_* will decrease $U_{\mathrm{post}}^{*}$ in this case. Dividing both sides of equation (21) by *U_pre_* and using the definition of *c_syn_*,

(22) $$\frac{U_{\mathrm{post}}^{*}}{U_{\mathrm{pre}}}=c_{\mathrm{syn}}=\frac{\frac{g_{s}}{R}\cdot U_{\mathrm{pre}}\cdot \Delta E_{s}+I_{\mathrm{app}}}{U_{\mathrm{pre}}\cdot \left(1+\frac{g_{s}}{R}\cdot U_{\mathrm{pre}}\right)}.$$

As in the previous section, we will solve for *c_syn_* when *U_pre_* = *R* to simplify analysis. Making this substitution and reducing *R*/*R* terms,

(23) $$c_{\mathrm{syn}}\cdot R=\frac{g_{s}\cdot \Delta E_{s}+R}{1+g_{s}}.$$

Multiplying both sides by the denominator of the right hand side and expanding,

(24) $$c_{\mathrm{syn}}\cdot R+c_{\mathrm{syn}}\cdot R\cdot g_{s}=g_{s}\cdot \Delta E_{s}+R.$$

Collecting *g_s_* terms on the left hand side,

(25) $$c_{\mathrm{syn}}\cdot R\cdot g_{s}-g_{s}\cdot \Delta E_{s}=R-c_{\mathrm{syn}}\cdot R.$$

Solving equation (25) for *g_s_*,

(26) $$g_{s}=\frac{c_{\mathrm{syn}}\cdot R-R}{\Delta E_{s}-c_{\mathrm{syn}}\cdot R}.$$

Just as in Sec. 3.1, *g_s_* > 0 depends only on *R*, which the designer specifies beforehand, Δ*E_s_*, which is limited by biological constraints, and *c_syn_*, which the designer picks based on network function. Δ*E_s_* should be negative, and as close to 0 as possible to minimize hyperpolarization of the postsynaptic neuron. Equation (26) will be used to tune division and multiplication networks (Secs. 3.5 and 3.6).

### 3.3 Addition

A subnetwork that approximates linear addition of the form $U_{\mathrm{post}}^{*}=k_{\mathrm{syn}}\cdot \left(U_{\mathrm{pre},1}+U_{\mathrm{pre},2}\right)$ may underlie positive feedback mechanisms, which increase motor neuron activation proportional to sensory inputs such as force sensing organs (Zill et al., 2004), or used to sum sensory signals from different body segments (Mittelstaedt, 1957). We construct such a network by using two Signal Transmission pathways as presented in Sec. 3.1.

Let us rewrite equation (13) here, for clarity:

(27) $$U^{*}=\frac{\sum_{i=1}^{n}\frac{g_{s,i}}{R}\cdot U_{\mathrm{pre},i}\cdot \Delta E_{s,i}+I_{\mathrm{app}}}{1+\sum_{i=1}^{n}\frac{g_{s,i}}{R}\cdot U_{\mathrm{pre},i}}.$$

**Figure 2:**
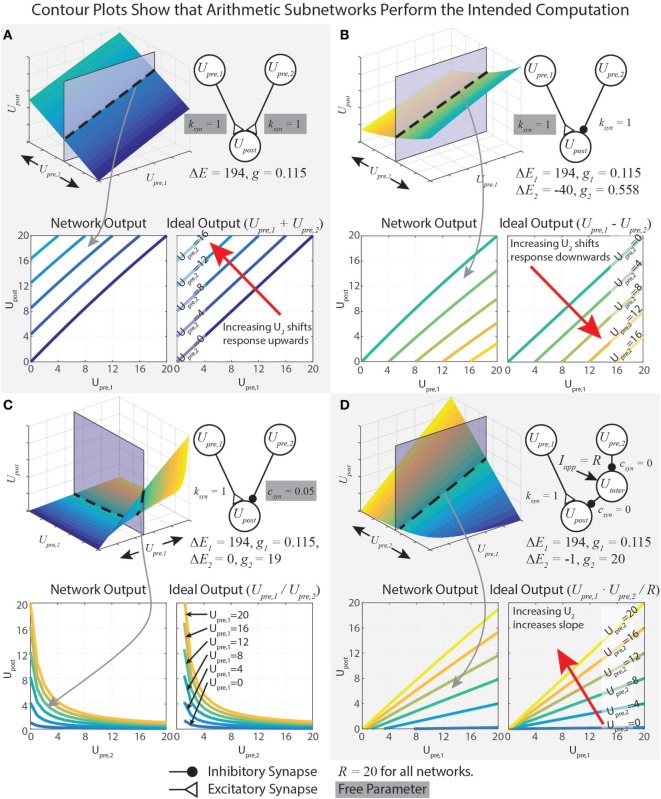
Data demonstrating the function of arithmetic networks. Each contour plot represents cross sections of the response surface, as depicted at the top. The network diagram, relevant parameters, and data are shown for addition **(A)**, subtraction **(B)**, division **(C)**, and multiplication **(D)**. Triangular synaptic terminations stand for excitatory inputs, and filled round terminations stand for inhibitory inputs. For each operation, the contour on the right is the ideal output, and the contour on the left is the actual operation for the parameter values listed. Free parameters from Table 1 are highlighted in gray.

This equation shows *U_pre,i_* in both the numerator and denominator. To capture addition, we wish to minimize the impact of *U_pre,i_* on the denominator. This is accomplished by minimizing *g_s_*. However, if *g_s_* = 0, then the network will not function at all. Therefore, we instead maximize Δ*E_s_*, which yields a small *g_s_* (equation (18)). Mathematically, there is no limit on Δ*E_s_*, but synaptic potentials are limited in biological systems. In our work, we choose the reversal potential of calcium (*E_s_* = 134 mV), which yields Δ*E_s_* = *E_s_* − *E_r_* = 134 − (−60) = 194 mV, and specify *R* = 20 mV. To design a pathway where *k_syn_* = 1, for example, we plug these values into equation (18), which gives *g_s_* = 115 nS. The contour plots in Figure 2A show that the network matches the ideal behavior very closely over the operating range *U_sum_* ∈ [0, *R*]. These design constraints are summarized in Table 1.

### 3.4 Subtraction

A subnetwork that approximates linear subtraction of the form $U_{\mathrm{post}}^{*}=k_{\mathrm{syn}}\cdot \left(U_{\mathrm{pre},1}-U_{\mathrm{pre},2}\right)$ may underlie negative feedback mechanisms, which are important for controlling many parameters in locomotion (Pearson, 1993; Peterka, 2003; Buschmann et al., 2015). Just as in the previous section, equation (18) is used to find *g_s_* for each pathway.

Designing a subtraction network requires that we pay attention to how the two synapses affect one another. Since the reversal potentials of hyperpolarizing ion channels are not much more negative than typical resting potentials, larger *g*_*s*,2_ values are required to transmit information than for depolarizing ion channels. This makes it harder to minimize *g_s_* like we did in the previous section. Equation (13) enables us to constrain *g*_*s*,2_ such that when *U*_*pre*,1_ = *R* and *U*_*pre*,2_ = *R*, $U_{\mathrm{post}}^{*}\;=0$. Starting with the neuron response in equation (13) for two synaptic currents and no applied current,

(28) $$U_{\mathrm{post}}^{*}=\frac{g_{s,1}∕R\cdot U_{\mathrm{pre},1}\cdot \Delta E_{s,1}+g_{s,2}∕R\cdot U_{\mathrm{pre},2}\cdot \Delta E_{s,2}}{1+g_{s,1}∕R\cdot U_{\mathrm{pre},1}+g_{s,2}∕R\cdot U_{\mathrm{pre},2}}.$$

Substituting in *U*_*pre*,1_ = *R, U*_*pre*,2_ = *R*, and $U_{\mathrm{post}}^{*}=0$,

(29) $$0=\frac{g_{s,1}∕R\cdot R\cdot \Delta E_{s,1}+g_{s,2}∕R\cdot R\cdot \Delta E_{s,2}}{1+g_{s,1}∕R\cdot R+g_{s,2}∕R\cdot R}$$

(30) $$0=\frac{g_{s,1}\cdot \Delta E_{s,1}+g_{s,2}\cdot \Delta E_{s,2}}{1+g_{s,1}+g_{s,2}}$$

(31) $$0=g_{s,1}\cdot \Delta E_{s,1}+g_{s,2}\cdot \Delta E_{s,2}$$

(32) $$g_{s,2}=\frac{\Delta E_{s,1}}{\Delta E_{s,2}}\cdot -g_{s,1}.$$

Substituting equation (18) for *g*_*s*,1_,

(33) $$g_{s,2}=\frac{\Delta E_{s,1}}{\Delta E_{s,2}}\cdot \frac{-k_{\mathrm{syn}}\cdot R}{\Delta E_{s,1}-k_{\mathrm{syn}}\cdot R}.$$

To be physically realizable, *g*_*s*,2_ > 0. Because *g*_*s*,1_ > 0 and Δ*E*_*s*,1_ > 0, *g*_*s*,2_ > 0 if and only if Δ*E*_*s*,2_ < 0. Thus, it is critical that Δ*E*_*s*,2_ < 0.

Just as for the addition network, we minimize *g*_*s*,1_ by maximizing Δ*E*_*s*,1_. If *R* = 20 mV and *k_syn_* = 1, then *g*_*s*,1_ = 115 nS and Δ*E*_*s*,1_ = 194 mV. To tune *g*_*s*,2_, we first select Δ*E*_*s*,2_ = −40 mV, then we solve equation (33) to find *g*_*s*,2_ = 558 nS. These design constraints are summarized in Table 1, and Figure 2B graphically shows the accuracy of the subtraction network.

### 3.5 Division

A subnetwork that approximates division of the form

(34) $$U_{\mathrm{post}}^{*}=\frac{U_{\mathrm{pre},1}}{1+\frac{1-c_{\mathrm{syn}}}{c_{\mathrm{syn}}\cdot R}\cdot U_{\mathrm{pre},2}}$$

replicates the function of GABA synapses that regulate activity in the brain. A key reason for this behavior is that the reversal potential of GABA-ergic synapses is about equal to the resting potential of the postsynaptic neuron (Trappenberg, 2009). Equation (26) is used to find *g_s_* for the division pathway.

The synapse from *U*_*pre*,1_ to *U_post_* is tuned as an excitatory Signal Transmission pathway with *k* = 1, as in Sec. 3.1. In our work, *R* = 20 mV, Δ*E*_*s*,1_ = 194 mV, and equation (18) tells us that *g*_*s*,1_ = 115 nS. Such a small *g_s_* ensures that the signal from *U*_*pre*,1_ to *U_post_* is transmitted without greatly affecting the sensitivity of *U_post_* to inputs. That is, the effect of *U*_*pre*,1_ on the denominator of $U_{\mathrm{post}}^{*}$ is very nearly 0.

The synapse from *U*_*pre*,2_ to *U_post_* is tuned as a Signal Modulation pathway, as analyzed in Sec. 3.2. Setting Δ*E*_*s*,2_ = 0 will eliminate *U*_*pre*,2_’s influence on the numerator of $U_{\mathrm{post}}^{*}$. Substituting this case into equation (26) and reducing,

(35) $$g_{s,2}=\frac{1-c_{\mathrm{syn}}}{c_{\mathrm{syn}}},$$

where $U_{\mathrm{post}}^{*}=c_{\mathrm{syn}}\cdot R$ when *U*_*pre*,1_ = *U*_*pre*,2_ = *R*, their maximal value. Equation (35) also reveals that since *g*_*s*,2_ > 0, 0 < *c_syn_* < 1.

The steady-state response of the network is the result of these two synaptic inputs, as written in equation (28). Substituting equation (35), and specifying that *k*_*syn*,1_ = 1, $U_{\mathrm{post}}^{*}$ simplifies to

(36) Upost∗=gs,1∕R⋅ΔEs,1⋅1Upre,1+gs,2∕R⋅ΔEs,2⋅0Upre,21+gs,1⋅0Upre,1∕R+1−csyncsyn⋅Upre,2∕R≈Upre,11+1−csyncsyn⋅R⋅Upre,2

In our network, we wished $U_{\mathrm{post}}^{*}=1$ when *U*_*pre*,2_ = *R*, so we set *c_syn_* = 1/*R* = 0.05, which makes *g*_*s*,2_ = 19 μS. When *c_syn_* is close to 0, *U*_*pre*,2_ can strongly reduce *U_post_*’s sensitivity to inputs. When *c_syn_* is close to 1, *U*_*pre*,2_ can only weakly reduce *U_post_*’s sensitivity to inputs. Figure 2C shows that this network performs the intended division of the signals. Table 1 summarizes these design constraints.

### 3.6 Multiplication

A subnetwork that approximates multiplication of the form $U_{\mathrm{post}}^{*}=U_{\mathrm{pre},1}\cdot U_{\mathrm{pre},2}∕R$ can be used to control the gain of a sensory feedback loop, a frequently observed characteristic of neural systems that control locomotion (Cruse, 1981; Gabriel and Büschges, 2007) and posture (Peterka and Loughlin, 2004).

A multiplication network can be assembled by replacing the Modulatory Pathway in the division network with two identical Modulatory Pathways in series, connected into a disinhibitory network (see Figure 2D). This works because the product of two numbers, *a*⋅*b* = *a*/(1/*b*). However, tuning the Modulatory Pathway for the multiplication network differs from tuning the division network. This is because the right-side pathway of the network in Figure 2D must make $U_{\mathrm{post}}^{*}=0$, no matter how active *U*_*pre*,1_ becomes (because *a*⋅0 = 0, no matter the value of *a*). Thus, according to equation (22), *c_syn_* = 0, unlike the division network, for which 0 < *c_syn_* < 1. Solving equation (26) when *c_syn_* = 0 reveals that

(37) $$g_{s,2}=-R∕\Delta E_{s,2}.$$

To solve for *g*_*s*,2_, we must first select Δ*E*_*s*,2_. If Δ*E*_*s*,2_ = 0 like for the division network, then equation (37) divides by 0. If Δ*E*_*s*,2_ > 0, then *g*_*s*,2_ < 0, which is physically not realizable. Therefore, we must choose a value Δ*E*_*s*,2_ < 0. The more negative Δ*E*_*s*,2_ is, the more small-amplitude signals are clipped; however, the less negative it is, the larger *g*_*s*,2_ must be. Therefore, *g*_*s*,2_ is the limiting factor to maintain biological realism. We have chosen *g*_*s*,2_ = 20 μS and *R* = 20 mV, making Δ*E*_*s*,2_ = −1.

Now that we have designed one of the Modulatory synapses, we can calculate the response of the complete multiplication network seen in Figure 2D, which includes two identical Modulatory Pathways in series. When *U*_*pre*,2_ is inactive, then it does not inhibit *U_inter_*, which is tonically active. In this case, *U_inter_*’s activity completely desensitizes *U_post_* to inputs. When *U*_*pre*,2_ is active, then it inhibits *U_inter_*. In this case, *U_inter_* is hyperpolarized, and cannot desensitize *U_post_* to inputs. To show that this is the case, let us find the full response of the system. We first calculate $U_{\mathrm{inter}}^{*}$, which has one Modulatory Pathway input and a tonic applied current *I_app_* = *R*. Its response is the same as in equation (21), with the constraint from equation (37), which causes terms to cancel:

(38) $$U_{\mathrm{inter}}^{*}=\frac{\frac{g_{s,2}}{R}\cdot U_{\mathrm{pre},2}\cdot -\frac{R}{g_{s,2}}+R}{1-\frac{U_{\mathrm{pre},2}}{\Delta E_{s,2}}}=\frac{R-U_{\mathrm{pre},2}}{1-\frac{U_{\mathrm{pre},2}}{\Delta E_{s,2}}}.$$

*U_post_* has two presynaptic neurons, *U*_*pre*,1_ and *U_inter_*. The synapse from *U*_*pre*,1_ is a Signal Transmission synapse, and the synapse from *U_inter_* is a Signal Modulation synapse. Its response is found via equation (13),

(39) $$U_{\mathrm{post}}^{*}=\frac{\frac{g_{s,3}}{R}\cdot U_{\mathrm{inter}}\cdot \Delta E_{s,3}+\frac{g_{s,1}}{R}\cdot U_{\mathrm{pre},1}\cdot \Delta E_{s,1}}{1+\frac{g_{s,3}}{R}\cdot U_{\mathrm{inter}}+\frac{g_{s,1}}{R}\cdot U_{\mathrm{pre},1}}.$$

We showed in Sec. 3.3 that equation (18) can be used to design a synapse that transmits the presynaptic neuron’s activity to the postsynaptic neuron, while minimizing its impact on the denominator of the postsynaptic neuron’s steady-state response, $U_{\mathrm{post}}^{*}$. This enables us to approximate *U*_*pre*,1_’s effect on $U_{\mathrm{post}}^{*}$ as an applied current *I_app_* ≈ *U*_*pre*,1_. Making this substitution in equation (39),

(40) $$U_{\mathrm{post}}^{*}\approx \frac{\frac{g_{s,3}}{R}\cdot U_{\mathrm{inter}}\cdot \Delta E_{s,3}+U_{\mathrm{pre},1}}{1+\frac{g_{s,3}}{R}\cdot U_{\mathrm{inter}}}.$$

Because we previously specified that the Modulatory Pathways are identical, we can apply the constraint from equation (37),

(41) $$U_{\mathrm{post}}^{*}=\frac{U_{\mathrm{pre},1}-U_{\mathrm{inter}}}{1-\frac{U_{\mathrm{inter}}}{\Delta E_{s,3}}}.$$

We can now substitute equation (38) for *U_inter_*,

(42) $$U_{\mathrm{post}}^{*}=\frac{U_{\mathrm{pre},1}-\frac{R-U_{\mathrm{pre},2}}{1-\frac{U_{\mathrm{pre},2}}{\Delta E_{s,2}}}}{1-\frac{1}{\Delta E_{s,3}}\cdot \frac{R-U_{\mathrm{pre},2}}{1-\frac{U_{\mathrm{pre},2}}{\Delta E_{s,2}}}}.$$

This expression can be simplified. First, as noted previously, synapses 2 and 3 are identical, so Δ*E*_*s*,2_ = Δ*E*_*s*,3_ = Δ*E_s_*. Second, we can multiply the first term in both the numerator and denominator by the factor (1 − *U*_*pre*,2_/Δ*E_s_*), which enables us to combine terms. Performing these simplifications,

(43) $$U_{\mathrm{post}}^{*}=\frac{U_{\mathrm{pre},1}-U_{\mathrm{pre},1}\cdot U_{\mathrm{pre},2}∕\Delta E_{s}-R+U_{\mathrm{pre},2}}{1-U_{\mathrm{pre},2}∕\Delta E_{s}-R∕\Delta E_{s}+U_{\mathrm{pre},2}∕\Delta E_{s}},$$

(44) $$U_{\mathrm{post}}^{*}=\frac{-U_{\mathrm{pre},1}\cdot U_{\mathrm{pre},2}∕\Delta E_{s}+U_{\mathrm{pre},1}+U_{\mathrm{pre},2}-R}{1-R∕\Delta E_{s}}.$$

Equation (44) contains a lot of information about how the multiplication network functions. First, *U_post_*’s response indeed contains a term that multiplies *U*_*pre*,1_ and *U*_*pre*,2_. When Δ*E_s_* = −1, then $U_{\mathrm{post}}^{*}$scales with *U*_*pre*,1_⋅*U*_*pre*,2_ in a 1:1 fashion. Second, the numerator will be ≤ 0 if either *U*_*pre*,1_ = 0 or *U*_*pre*,2_ = 0, $U_{\mathrm{post}}^{*}\leq 0$. This is because *U*_*pre*,1_ and *U*_*pre*,2_ must each be less than or equal to *R*. If either input is greater than *R*, then their synaptic inputs to *U_post_* will saturate (see equation (4)), preventing this condition from being violated. Third, the denominator does not depend on the input values. Technically, because of the approximation made in equation (40), the denominator does change slightly with *U*_*pre*,1_. However, with our chosen values of *R* (20), Δ*E_s_* (−1), and *g*_*s*,1_ (0.115), this change is less than 1%, justifying this approximation. Figure 2D demonstrates that this network multiplies the two inputs.

Table 1 summarizes the function, component pathways, constraint equations, and free parameters of each network from this section. This analysis enables direct construction and parameter selection for functional subnetworks that can be assembled into more complex networks capable of performing real-time robotic control (e.g., Szczecinski and Quinn (2017) and Szczecinski et al. (2017a)). Additionally, one of the key advantages to using dynamic neural systems for motor control is the handling of time varying signals. The next section examines how the dynamics of these neurons can be exploited to perform calculus on signals.

## 4 Methods: Dynamic Networks

The differential equation for a single neuron’s response (equation (1)) can be solved analytically. Solving an equation *dx*/*dt* = *f* (*x*) is simplified if the equilibrium state is x* = 0, so as in Sec. 3, the substitution *U* = *V* − *E_r_* is made. Additionally, the membrane conductance *G_m_* and capacitance *C_m_* can be combined into a new parameter τ = *C_m_*/*G_m_*, which is a more intuitive parameter when discussing dynamic networks. This section uses analysis from the previous section, plus additional analysis, to derive design constraints for networks that differentiate or integrate input signals over time.

### 4.1 Differentiation

One dynamic response neural systems are known to utilize is differentiation of signals. Early examination of neural networks led to the discovery of the Reichardt detector network (Reichardt, 1961), an autocorrelation network with delays that approximates the differential of an incoming signal. Other examples include human balance, which relies on feedback proportional to the position, velocity, and acceleration of the center of mass (Peterka, 2003; Safavynia and Ting, 2012). Also, positive velocity feedback plays an important role in insect muscle control (Cruse, 1981).

**Figure 3:**
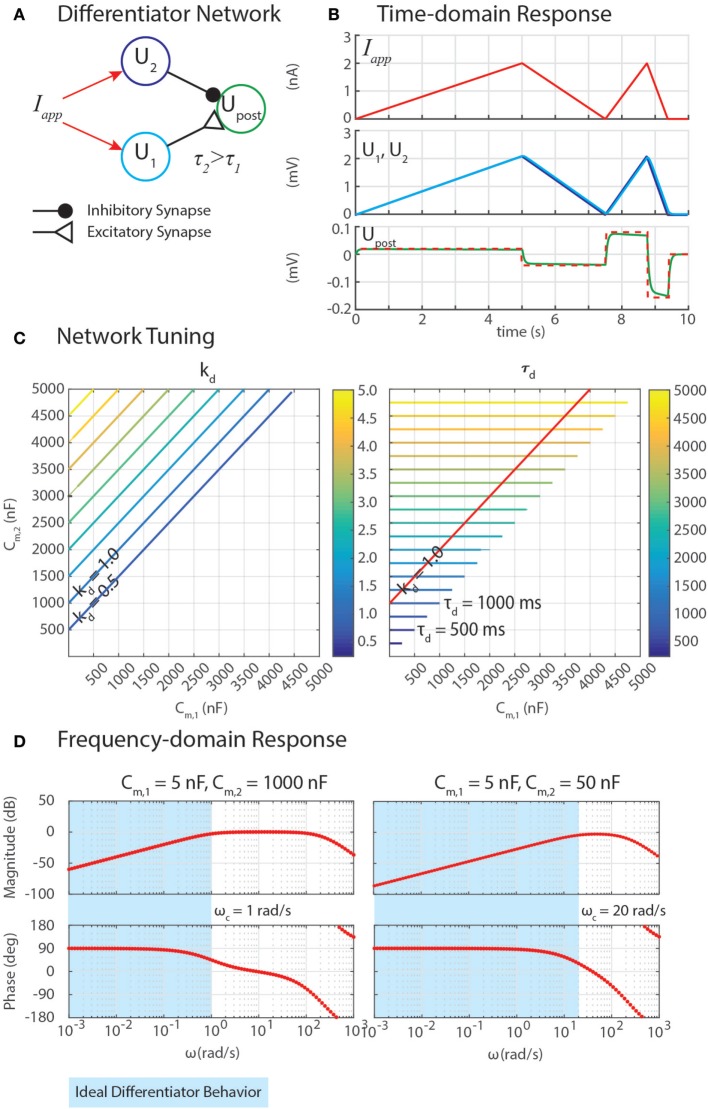
**(A)** A network can exploit neural dynamics to compute the differential of an incoming signal. **(B)** When given an applied current in the form of ramps, the network returns steps whose heights are proportional to the slopes of the ramps. **(C)** The amplification of the differential, *k_d_*, and the time constant of the network, τ*_d_*, depend on the capacitance of the neurons, *C*_*m*,1_ and *C*_*m*,2_. **(D)** Frequency domain analysis enables the identification of the cutoff frequency *ω_c_*, enabling the network to naturally filter out high-frequency noise.

We have developed differentiation networks based on the Reichardt detector network, shown in Figure 3A. We can understand its function by examining a neuron’s response to a ramp input, *I_app_* = *A*⋅*t*, where *A* is an arbitrary slope of the ramp. The response of the network should be a step with a magnitude proportional to *A*, as shown in Figure 3B. Inserting this applied current into equation (6), a single neuron’s response is

(45) $$C_{m}\cdot \frac{\mathrm{dU}}{\mathrm{dt}}=-U+A\cdot t$$

(46) $$C_{m}\cdot \frac{\mathrm{dU}}{\mathrm{dt}}+U=A\cdot t.$$

The response of the neuron, *U*(*t*), is the sum of the particular and homogeneous solutions to equation (46), *U_p_*(*t*) and *U_h_*(*t*), respectively. Simulating the dynamics of equation (46) suggests that the particular solution is a ramp of slope *A*, which lags behind the input with a time constant *C_m_*. To confirm this, we can substitute a candidate solution and its derivative into equation (46), and check for equality. The result is the particular (i.e., steady-state) response,

(47) $$U_{p}\left(t\right)=A\cdot \left(t-C_{m}\right)$$

This means that if the same *I_app_* were injected into neurons with different *C*_*m*_ values, and then their outputs were subtracted from one another with a network from Sec. 3.4, the network would perform a finite-difference approximation of the derivative of *I_app_*, once the transient response decays (illustrated in Figures 3A,B).

Calculating the homogeneous solution, *U_h_*(*t*), informs us how quickly the transient response decays. The homogeneous solution to first-order linear equation like equation (46) is well-known, *U_h_*(*t*) = *b*⋅exp(− *t*/*C_m_*). The constant *b* is found by plugging the initial condition into the full response, *U*(*t*) = *U_p_*(*t*) + *U_h_*(*t*),

(48) $$b=A\cdot C_{m}.$$

To tune this network, the response of *U_post_* is written as the difference between neuron *U*_*pre*,1_ with *C*_*m*,1_ and neuron *U*_*pre*,2_ with *C*_*m*,2_ > *C*_*m*,1_,

(49) $$\begin{array}{ll} U_{\mathrm{post}}\left(t\right) & =U_{\mathrm{pre},1}\left(t\right)-U_{\mathrm{pre},2}\left(t\right)\\ & =A\cdot t-A\cdot C_{m,1}\cdot \left(1-\exp\left(-t∕C_{m,1}\right)\right)-\left(A\cdot t-A\cdot C_{m,2}\cdot \left(1-\exp\left(-t∕C_{m,2}\right)\right)\right). \end{array}$$

Canceling the terms that are linear in *t* and expanding,

(50) $$U_{\mathrm{post}}\left(t\right)=A\cdot \left(C_{m,2}-C_{m,1}\right)+A\cdot \left(C_{m,1}\cdot \exp\left(-t∕C_{m,1}\right)-C_{m,2}\cdot \exp\left(-t∕C_{m,2}\right)\right).$$

Properly tuning a differentiator network requires tuning *C*_*m*,1_ and *C*_*m*,2_ to obtain the intended gain of the network, *k_d_*, and an appropriately high cutoff frequency, *ω_c_*. Equation (50) reveals how these may be tuned. First, the steady-state response of this network to a ramp input defines *k_d_* = (*C*_*m*,2_ − *C*_*m*,1_). Second, the cutoff frequency *ω_c_* = 1/τ*_d_* quantifies the frequency of incoming signals (i.e., *I_app_* = *A*⋅sin(*ω*⋅*t*)) above which the network’s response has less than half the energy of a lower-frequency signal. This is especially useful because although differential calculations amplify high-frequency noise, this network filters out noise with a frequency *ω* > *ω_c_*. Because *C*_*m*,2_ > *C*_*m*,1_, the time constant τ*_d_* = *C*_*m*,2_.

Figure 3C shows contours of *k_d_* and τ*_d_* as *C*_*m*,1_ and *C*_*m*,2_ change. The plots show that increasing *C*_*m*,2_ relative to *C*_*m*,1_ increases *k_d_*, which may be valuable for amplifying signals. However, this also increases τ*_d_*, making *ω_c_* impractically low, which will cause the network’s output to lag behind the input substantially. The contour for *k_d_* = 1 is drawn on the contour of τ*_d_*, showing that the smallest τ*_d_* achievable for this gain value is 1,000 ms, which would filter out all incoming signals for which *ω* > *ω_c_* = 1/(1 *s*) = 1 rad/s (0.159 Hz).

**Figure 4:**
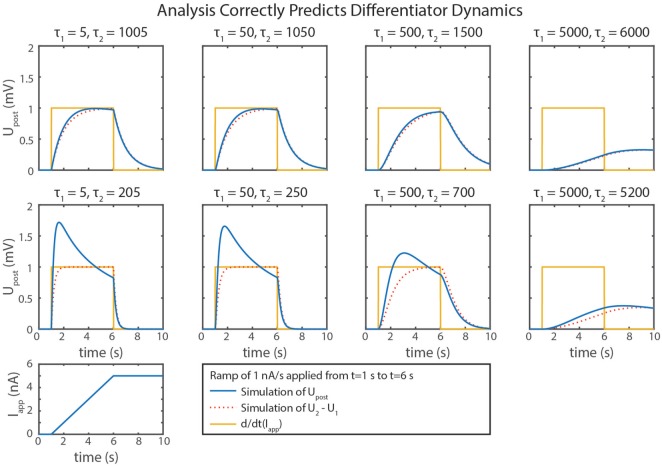
Simulation data from eight trials with the differentiator network are shown. Different values of *C*_*m*,1_ and *C*_*m*,2_ were used in each. $U_{\mathrm{post}}^{*}$ is plotted in blue, *U*_2_ − *U*_1_ is plotted in dotted red, and the actual rate of change of the input, *d*/*dt*(*I_app_*), is plotted in gold.

We can gain further insight into tuning τ*_d_* using our FeedbackDesign tool (Szczecinski et al., 2017b). Figure 3D shows Bode plots for this network’s response, given two different values for *C*_*m*,2_. When *C*_*m*,2_ = 1,000 nF, like in Figure 3B, the network functions properly for inputs with *ω* < 1 rad/s, as predicted in the previous paragraph. Lowering *C*_*m*,2_ to 50 nF increases *ω_c_* to 20 rad/s (3.18 Hz). Lowering *C*_*m*,2_ also lowers the magnitude response as a function of *ω*, that is, it decreases *k_d_*. To regain this lost gain, we may increase *k_syn_* in the subtraction network. Figure 4 shows simulation data that explores this tradeoff. Table 2 lists how to use τ*_d_* and *k_d_* to tune the entire differentiation network.

### 4.2 Integration

Our neuron model is a leaky integrator, which means that the membrane voltage will integrate an applied current, but “leak” current to return to its resting potential. As a result, data cannot be stored in individual neurons, because neurons only have one stable equilibrium point. A network that is constructed to have a marginally stable equilibrium curve (or subspace) will not leak. A network will have this property if the determinant of the Jacobian matrix is 0, or in other words, if it is not full rank (Khalil, 2002). Instead of leaking, it will maintain its activation when no external currents are applied; when currents are applied, the state of the system will change continuously. This is analogous to the position of a box on a table with friction; it will remain wherever it is placed indefinitely, unless an external force is applied. In this section, we expand on previous work (Szczecinski et al., 2017a) to show how to construct a network that is marginally stable by applying constraints to reduce the rank of its Jacobian matrix; demonstrate that such a network can be used to integrate signals over time; and relate the integration rate, *k_i_*, to the parameter values of the network, such that $\dot{U_{1}}=k_{i}\cdot I_{\mathrm{app}}$.

**Figure 5:**
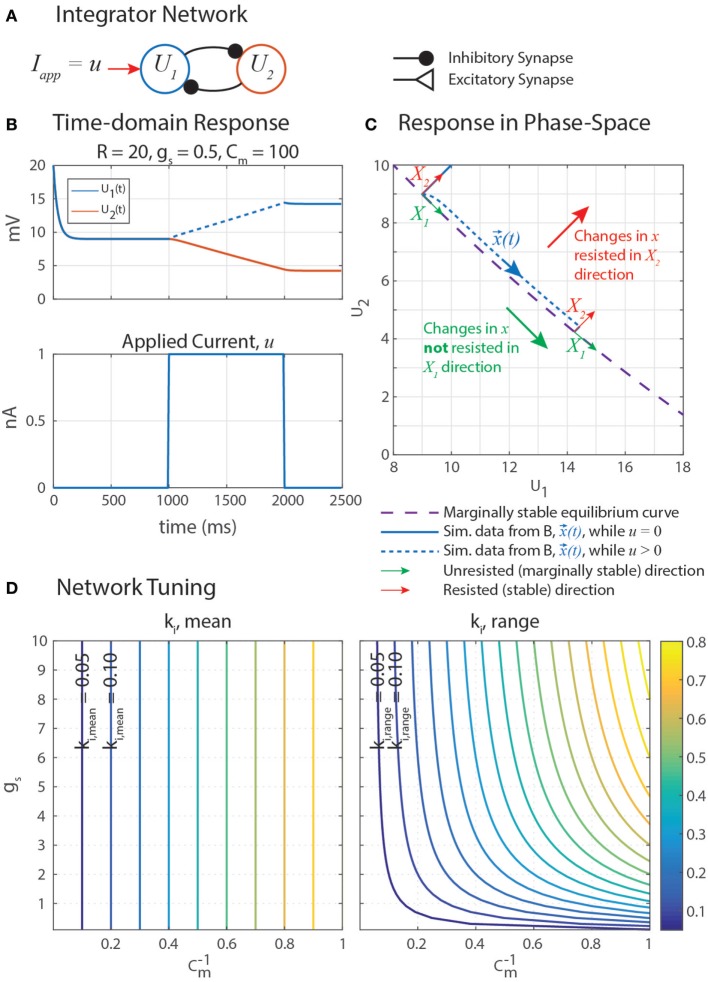
**(A)** A disinhibitory network can exploit neural dynamics to compute the integral of an incoming signal. **(B)** When given an applied current in the form of a step, the network response is a ramp whose slope is proportional to the amplitude of the step. **(C)** A plot of this data in the (*U*_1_, *U*_2_) phase space shows that when stimulated by applied current *u*, the system state, *x*(*t*) = [*U*_1_(*t*), *U*_2_(*t*)]*^T^* (blue), moves in the *X*_1_ direction (green) while maintaining a constant distance from the equilibrium subspace (dashed violet) in the *X*_2_ direction (red). This difference in behavior in each direction is because the eigenvalue associated with eigenvector *X*_1_, λ_1_ = 0, and the eigenvalue associated with eigenvector *X*_2_, λ_2_ < 0. *X*_1_ and *X*_2_ are drawn in multiple places because they depend on *x*(*t*), as shown in Appendix. **(D)** The mean rate of integration, *k*_*i*,mean_ (left), and the range of the rate of integration, *k*_*i*,range_ (right), depend on the synaptic conductance of mutual inhibition, *g_s_*, and the membrane capacitance of the neurons, *C_m_*. Note that the *x*-axis of these plots are 1/*C_m_*, to better space the contour lines.

Marginally stable networks are hypothesized to play an important role in navigation (Haferlach et al., 2007) and the regulation of muscle forces in posture (Lévy and Cruse, 2008). Some memory models use carefully tuned self-excitation to cancel the leak current with excitatory synaptic current (Seung et al., 2000). In a similar vein, our network uses self-disinhibition (Figure 5A) to produce a line-attractor network in which a continuum of marginally stable equilibrium states exist. Simulation data in Figure 5B shows that stimulating *U*_1_ with an applied constant current *u* causes *U*_1_ to increase at an apparently constant rate, and when *u* is removed, neither *U*_1_ nor *U*_2_ leak to their rest potentials. This is the behavior of an integrator, as described in the previous paragraph.

Let us write the response of the integrator network as shown in Figure 5A to find its equilibrium states. Each neuron has leak current, synaptic current, and a constant applied current. Let all parameter values be symmetrical between the two neurons. We make the same substitutions as before; *U* = *V* − *E_r_, E_r_* = *E_lo_*, Δ*E_s_* = *E_s_* − *E_r_*, and *R* = *E_hi_* − *E_lo_*. If *I_app_* = *R*,

(51) $$C_{m}\cdot \frac{{\mathrm{dU}}_{1}}{\mathrm{dt}}=-U_{1}+g_{s}\cdot \frac{U_{2}}{R}\cdot \left(\Delta E_{s}-U_{1}\right)+R$$

(52) $$C_{m}\cdot \frac{{\mathrm{dU}}_{2}}{\mathrm{dt}}=-U_{2}+g_{s}\cdot \frac{U_{1}}{R}\cdot \left(\Delta E_{s}-U_{2}\right)+R.$$

Moving dynamical terms to the left hand side, and applied current to the right hand side,

(53) $$\frac{{\mathrm{dU}}_{1}}{\mathrm{dt}}+\frac{1}{C_{m}}\left(U_{1}-g_{s}\cdot \frac{U_{2}}{R}\cdot \left(\Delta E_{s}-U_{1}\right)\right)=\frac{R}{C_{m}}$$

(54) $$\frac{{\mathrm{dU}}_{2}}{\mathrm{dt}}+\frac{1}{C_{m}}\left(U_{2}-g_{s}\cdot \frac{U_{1}}{R}\cdot \left(\Delta E_{s}-U_{2}\right)\right)=\frac{R}{C_{m}}$$

Solving equation (53) when *dU*_1_/*dt* = 0 reveals the equilibrium curve

(55) $$U_{2}=\frac{R\cdot \left(U_{1}-R\right)}{g_{s}\cdot \left(\Delta E_{s}-U_{1}\right)}.$$

Solving equation (54) when *dU*_2_/*dt* = 0 reveals the equilibrium curve

(56) $$U_{1}=\frac{R\cdot \left(U_{2}-R\right)}{g_{s}\cdot \left(\Delta E_{s}-U_{2}\right)},$$

which can be algebraically rearranged to be the same as equation (55) as long as *g_s_* and Δ*E_s_* are constrained such that

(57) $$g_{s}\cdot \Delta E_{s}=-R.$$

Multiplying both sides of equation (56) by the denominator of the right hand side, and expanding,

(58) $$g_{s}\cdot \Delta E_{s}\cdot U_{1}-g\cdot U_{1}\cdot U_{2}=R\cdot U_{2}-R^{2}.$$

Collecting multiples of *U*_2_ and applying equation (57),

(59) $$U_{2}=\frac{R\cdot \left(U_{1}-R\right)}{g_{s}\cdot \left(\Delta E_{s}-U_{1}\right)}.$$

Thus, equations (55) and (56) are the same equilibrium curve if *g_s_* and Δ*E_s_* satisfy equation (57). This curve, drawn on the phase-space diagram in Figure 5C, describes every equilibrium state that this network can have. In other words, a [*U*_1_, *U*_2_] pair is an equilibrium state of the system if and only if it satisfies equation (55). In the coming paragraph, we will use eigenvalue analysis to show that this network always functions as an integrator, as long as equation (57) is satisfied.

To find the system’s eigenvalues, let us write equations (53) and (54) together in matrix form,

(60) $$\left[\begin{array}{c} \dot{U_{1}}\\ \dot{U_{2}} \end{array}\right]+\frac{1}{C_{m}}\cdot \left[\begin{array}{cc} 1+U_{2}\cdot \frac{g_{s}}{R} & \;\;\;\;\frac{-g_{s}}{R}\cdot \left(\Delta E_{s}-U_{1}\right)\\ \frac{-g_{s}}{R}\cdot \left(\Delta E_{s}-U_{2}\right) & \;\;\;\;1+U_{1}\cdot \frac{g_{s}}{R} \end{array}\right]\cdot \left[\begin{array}{c} U_{1}\\ U_{2} \end{array}\right]=\frac{1}{C_{m}}\cdot \left[\begin{array}{c} R\\ R \end{array}\right]\;\;,$$

in which the square matrix is *J*, the system Jacobian. Because *J* contains *U*_1_ and *U*_2_ terms, it is not constant, but still describes the stability of the system, given specific values of *U*_1_ and *U*_2_. To construct a marginally stable equilibrium subspace for the network, we must show that *J* has insufficient rank (i.e., the rows are identical) when *U*_1_ and *U*_2_ are at equilibrium (i.e., equation (55) is satisfied). However, the rows are identical, no matter the values of *U*_1_ and *U*_2_, if we apply the constraint from equations (57) to (60),

(61) $$\left[\begin{array}{c} \dot{U_{1}}\\ \dot{U_{2}} \end{array}\right]+\frac{1}{C_{m}}\cdot \left[\begin{array}{cc} 1+\frac{g_{s}}{R}\cdot U_{2} & 1+\frac{g_{s}}{R}\cdot U_{1}\\ 1+\frac{g_{s}}{R}\cdot U_{2} & 1+\frac{g_{s}}{R}\cdot U_{1} \end{array}\right]\cdot \left[\begin{array}{c} U_{1}\\ U_{2} \end{array}\right]=\frac{1}{C_{m}}\cdot \left[\begin{array}{c} R\\ R \end{array}\right]\;\;.$$

Thus, the system will always have one null direction, and we do not need to calculate *J* for specific equilibrium conditions to determine the system’s stability. To make notation more compact, let us define

(62) $$a=1+g_{s}∕R\cdot U_{1}$$

(63) $$b=1+g_{s}∕R\cdot U_{2}$$

These expressions let us write equation (61) as simply

(64) $$\left[\begin{array}{c} \dot{U_{1}}\\ \dot{U_{2}} \end{array}\right]+\left[\begin{array}{cc} b∕C_{m} & a∕C_{m}\\ b∕C_{m} & a∕C_{m} \end{array}\right]\cdot \left[\begin{array}{c} U_{1}\\ U_{2} \end{array}\right]=\left[\begin{array}{c} R∕C_{m}\\ R∕C_{m} \end{array}\right]\;\;.$$

Plotting the simulation data of the network’s forced response from Figure 5B on a phase-space diagram (Figure 5C) suggests that *u* causes *U*_1_ and *U*_2_ to change in such a way that the state of the system ($\vec{x}\left(t\right)$, blue) moves *tangent to* the equilibrium curve (dashed violet), with some constant distance *away* from it. These curves do not overlap because the forced response is not the same as the equilibrium condition while the external current *u* is applied. Motion in the *X*_2_ direction is resisted by the neural dynamics, much how a spring resists the translation of an object with an applied force.

Nonetheless, these direction-dependent responses suggest that the state can be generalized into two decoupled degrees of freedom in the phase-space: unresisted, marginally stable motion parallel to the equilibrium curve (*X*_1_, green in Figure 5C); and resisted, stable motion away from the equilibrium curve (*X*_2_, red). The natural coordinates, $\vec{x}={\left[U_{1},U_{2}\right]}^{T}$, are transformed into generalized coordinates, $\vec{q}={\left[q_{1},q_{2}\right]}^{T}$, by a matrix *X* comprised of the eigenvectors of *J*. This same transformation matrix is used to transform *J* into the generalized coordinate system, yielding *J_q_*. *J_q_* is diagonal, decoupling the dynamics of the generalized coordinates and enabling us to quantify how quickly $\vec{x}$ moves parallel to the equilibrium curve.

Appendix shows the calculation of *X*, with *q*_1_ representing the marginally stable mode and *q*_2_ representing the stable mode. Using *X*, we can transform the system into generalized coordinates. First, we write the dynamics from equation (64) in a compact format.

(65) $$\dot{\vec{x}}+J\vec{x}=\vec{F},$$

where *J* is the square matrix in equation (64) and

(66) $$\vec{F}=\left[\begin{array}{c} R∕C_{m}+u∕C_{m}\\ R∕C_{m} \end{array}\right]\;\;.$$

The generalized coordinates, $\vec{q}$, are defined as

(67) $$\vec{x}=X\vec{q}.$$

To transform equation (65) into generalized coordinates, premultiply both sides of equation (65) by *X^−^*^1^,

(68) $$\dot{\vec{q}}+J_{q}\vec{q}=\vec{Q},$$

where *J_q_* = *X*^−1^*JX* and $\vec{Q}=X^{-1}\vec{F}$. The top and bottom rows of equation (68) are decoupled because *J_q_* is a diagonal matrix. Furthermore, $J_{q}^{i,i}=\lambda_{i}$, meaning that $J_{q}^{1,1}=0$, so the system simplifies even further.

To find the particular solution of this system, we can guess the form of *q*_*p*,1_ and *q*_*p*,2_, and substitute those in to equation (68). We observe that ${\dot{q}}_{1}\left(t\right)=B\cdot u$ in steady state, where *B* is a constant that relates ${\dot{q}}_{1}\left(t\right)$ and *u*. *q*_1_(*t*) would be the integral of ${\dot{q}}_{1}\left(t\right)$, but because the top row of *J_q_* is zeros, it will not appear in the particular solution, and thus need not be explicitly included. We also observe that ${\dot{q}}_{2}\left(t\right)=0$ in steady state, so *q*_2_(*t*) = *D*, a constant. We can calculate $\vec{Q}=X^{-1}F$ using *X*^−1^, which is calculated in Appendix (equation (A9)). Solving for the particular solution of this system, ${\dot{\vec{q}}}_{p}\left(t\right)$,

(69) $$\begin{array}{lll} {\dot{\vec{q}}}_{p}\left(t\right)+J_{q}\vec{q_{p}} & =\left[\begin{array}{c} B\cdot u\\ 0 \end{array}\right]+\left[\begin{array}{cc} 0 & 0\\ 0 & \frac{a+b}{C_{m}} \end{array}\right]\cdot \left[\begin{array}{c} q_{p,1}\\ D \end{array}\right] & =\left[\begin{array}{c} \frac{a\cdot d}{C_{m}\cdot \left(a+b\right)}\cdot u\\ \frac{R\sqrt{2}}{C_{m}}+\frac{b\sqrt{2}}{C_{m}\cdot \left(a+b\right)}\cdot u. \end{array}\right] \end{array}$$

(70) $$B=\frac{a\cdot d}{C_{m}\cdot \left(a+b\right)},$$

where *d* is defined in equation (A5). *B* describes how quickly *q*_*p*,1_ varies with *u*, but we want to know how quickly *U*_1_ varies with *u*. Therefore, we use equation (67) to transform ${\vec{\dot{q}}}_{p}={\left[B\cdot u,0\right]}^{T}$ into natural coordinates to find $\dot{\vec{x}}$,

(71) $$\dot{\vec{x_{p}}}=X\dot{\vec{q_{p}}}$$

(72) $$\begin{array}{ll} \left[\begin{array}{c} {\dot{U}}_{1,p}\left(t\right)\\ {\dot{U}}_{2,p}\left(t\right) \end{array}\right] & =\left[\begin{array}{cc} 1∕d & 1∕\sqrt{2}\\ -b∕\left(\mathrm{ad}\right) & 1∕\sqrt{2} \end{array}\right]\cdot \left[\begin{array}{c} \frac{a\cdot d}{C_{m}\cdot \left(a+b\right)}\cdot u\\ 0 \end{array}\right] \end{array}$$

(73) $${\dot{U}}_{1,p}\left(t\right)=\frac{a}{C_{m}\cdot \left(a+b\right)}\cdot u$$

(74) $$k_{i}=\frac{a}{C_{m}\cdot \left(a+b\right)}.$$

Recall that *a* and *b* are functions of *U*_1_ and *U*_2_, respectively. This means that *k_i_*, the integral gain of the network, is not a constant. To place bounds on *k_i_*, let us substitute equations (62) and (63) into equation (74),

(75) $$k_{i}=\frac{1+g_{s}∕R\cdot U_{1}}{C_{m}\cdot \left(2+g_{s}∕R\cdot \left(U_{1}+U_{2}\right)\right)}.$$

We can now plug in different values of *U*_1_ and *U*_2_ to see how *k_i_* varies. Using equations (55) and (56), we find that the most extreme cases are when [*U*_1_, *U*_2_] = [0, *R*] and [*U*_1_, *U*_2_] = [*R*, 0]. We can plug these cases into equation (75) to find the minimum and maximum values for *k_i_*,

(76) $$k_{i,\min}=\frac{1+g_{s}∕R\cdot 0}{C_{m}\cdot \left(2+g_{s}∕R\cdot \left(0+R\right)\right)}=\frac{1}{C_{m}\cdot \left(2+g_{s}\right)}$$

and

(77) $$k_{i,\max}=\frac{1+g_{s}∕R\cdot R}{C_{m}\cdot \left(2+g_{s}∕R\cdot \left(R+0\right)\right)}=\frac{1+g_{s}}{C_{m}\cdot \left(2+g_{s}\right)}.$$

The difference between *k*_*i*,min_ and *k*_*i*,max_:

(78) $$k_{i,\text{range}}=\frac{1+g_{s}}{C_{m}\cdot \left(2+g_{s}\right)}-\frac{1}{C_{m}\cdot \left(2+g_{s}\right)}=\frac{g_{s}}{C_{m}\cdot \left(2+g_{s}\right)}.$$

To find the mean rate of integration, we can calculate *k*_*i*,mean_ = (*k*_*i*,min_ + *k*_*i*,max_)/2,

(79) $$k_{i,\text{mean}}=\frac{1}{2\cdot C_{m}}.$$

This is the same value of *k_i_* obtained from computing *k_i_* when *U*_1_ = *U*_2_. This simple expression is a useful relationship for tuning the integrator network. One may select *C_m_* to obtain the intended mean integration rate, and then minimize the variation of the integration rate by minimizing *g_s_*, as long as equation (57) is satisfied.

**Figure 6:**
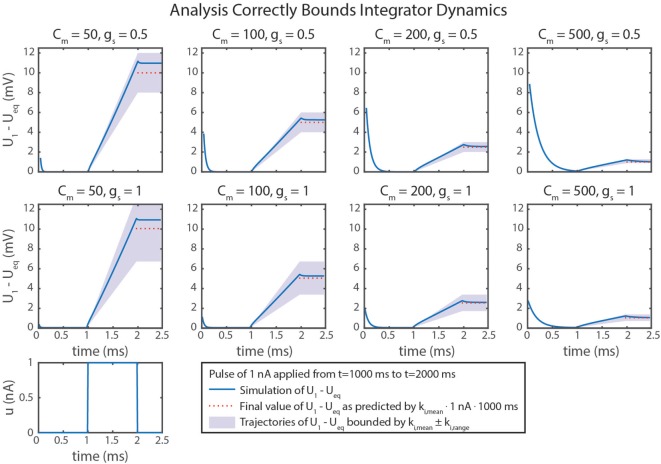
Simulation data from eight trials are shown. Different values of *C_m_* and *g_s_* were used in each. Neural dynamics are plotted as blue lines. The expected final values of the simulations are plotted in dotted red lines. Regions bounded by *k*_*i*,mean_ ± *k*_*i*,range_ are shaded in violet. In every case, the actual outcome is correctly bounded. As demonstrated mathematically in the text, *k*_*i*,mean_ only depends on *C_m_*. In addition, *k*_*i*,range_ depends on *g_s_*, leading to more variation in *k_i_*, as indicated by larger shaded areas.

Figure 5D graphically demonstrates how *k*_*i*,mean_ and *k*_*i*,range_ determine *C_m_* and *g_s_*. Just as in equation (79), *k*_*i*,mean_ is a function only of *C_m_*. Therefore, the contour only shows vertical lines. The value of *k*_*i*,range_ is minimized by decreasing either *g_s_* or $C_{m}^{-1}$ (i.e., increasing *C_m_*). Figure 6 shows simulation data of the integrator’s response to a step input with eight different parameter value combinations. In every case, the change in *U*_1_ is bounded by the values of *k*_*i*,mean_ and *k*_*i*,range_. As shown in Figure 5D, increasing *C_m_* decreases the integration rate, and increasing *g_s_* increases the variation in the integration rate.

Table 2 summarizes the design approach for this integrator network. The mean and range of the integration rate are free parameters that are determined by the intended network performance. Using these values and the constraint in equation (57), the neurons’ *C_m_* value and the synapses’ *g_s_* and Δ*E_s_* values can be fully specified.

## 5 Application to a Robot Controller

**Figure 7:**
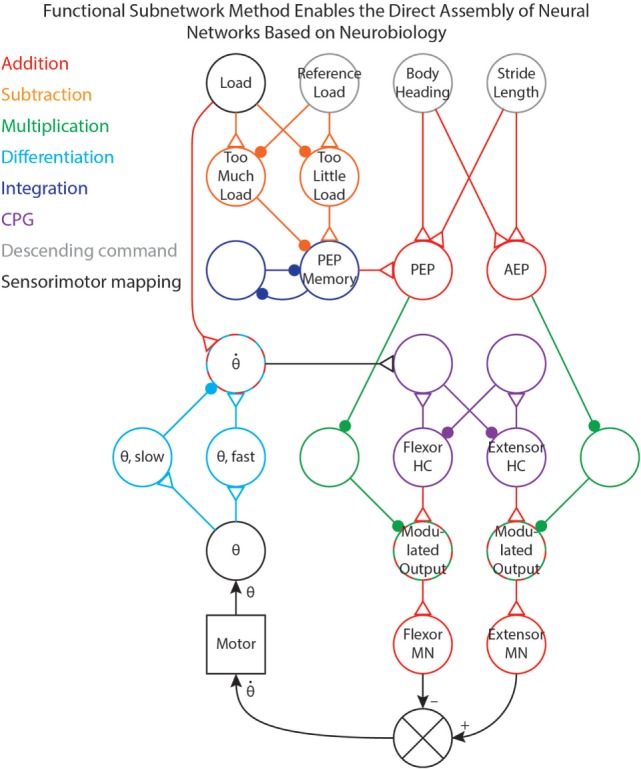
A simplified joint-control network from our previous work (Szczecinski and Quinn, 2017; Szczecinski et al., 2017a), with pathways color-coded based on the functional subnetwork.

We have used the methods in this paper to tune (i.e., select parameter values for) several different networks that control robotic stepping (Szczecinski and Quinn, 2017; Szczecinski et al., 2017a) and visual tracking (Szczecinski et al., 2017a). Once a network layout is determined, whether hypothetical or based on neurobiological findings, individual subnetworks can be identified and tuned to work together. Figure 7 shows a simplified joint-control network in which different functional pathways are color-coded. This illustrates how these functional subnetworks enable the direct assembly of control networks based on neurobiology. The neurobiological inspiration for these networks and the results of robotic experiments are presented in Szczecinski and Quinn (2017) and Szczecinski et al. (2017a), and so are omitted here.

The joint network in Figure 7 uses three simple descending commands (body heading, stride length, and reference leg load) to control the walking motion of one joint of a leg. The descending commands modulate the output of a central pattern generator (CPG) to control the speed of the motion, and sensory feedback is used to adjust both the timing and amplitude of motor output. Addition pathways are drawn in red. These include the mapping between body heading and stride length (i.e., descending commands, drawn in gray) to the PEP and AEP (Szczecinski and Quinn, 2017). The PEP can also be modulated by force feedback, which compares the load on the leg to a reference value (Szczecinski and Quinn, 2017, in review). This requires a subtraction network, drawn in orange, to compute if there is too much or too little load on the leg. The difference is used to adjust the PEP Memory network, which is an integration network, drawn in blue. This network adjusts the PEP over time, and remembers the motor command that produces the intended force.

The output of the CPG, drawn in purple, excites the motor neurons. Tuning CPG dynamics is discussed in our previous work (Szczecinski et al., 2017b). The PEP and AEP neurons adjust the motor output via multiplication pathways, drawn in green, which scale CPG output to the motor neurons based on the intended range of motion. Motor neuron activity controls the motor velocity, and the *θ* neuron receives position feedback from the motor via the mappings in Figure 1B. The motor velocity, computed by the cyan differentiation pathway, reinforces ongoing CPG behavior through the $\dot{\theta }$ neuron (Szczecinski et al., 2017b). A division pathway (not shown) can be used to normalize the velocity feedback to the joint’s commanded range of motion, simplifying the control of stepping frequency. The $\dot{\theta }$ neuron also receives some input from the Load neuron, ensuring that stance phase is stable (Szczecinski and Quinn, 2017, accepted). By using the functional subnetworks and the design constraints presented in this paper, we can rapidly and directly assemble models of neural systems that perform as intended without hand-tuning or optimization methods.

How are the “Free Parameters” in Tables 1 and 2 chosen? The free parameters fall into two classes: reversal potentials (i.e., Δ*E_s_*) and dynamical constants (e.g., *k*, τ, etc.). The reversal potentials are informed by biology. In this paper, we kept −40 < Δ*E_s_* < 194 mV (i.e., −100 < *E_s_* < 134 mV). The modeler could use reversal potentials from specific synapses if that data were available. The dynamical constants are informed by the function of the robot. For example, the *k_syn_* of the subtraction network in Figure 1B controls the stiffness of the controller, and may destabilize the system if not tuned to match the mechanical properties of the robot (Szczecinski et al., 2017b).

As another example, τ*_d_* and *k_d_* of the differentiator network in Figure 7 determines the robustness of CPG rhythms, and how well it entrains to sensory feedback (Szczecinski et al., 2017b). A slow, adaptively-walking robot may want a high *k_d_* to regularize CPG oscillations, whereas a fast running robot may want a low *k_d_* to be less sensitive to sensory feedback. Picking specific values for these free parameters ultimately depends on the intended behavior of the robot. The constraints in this paper enable the designer to think in terms of more traditional robotics quantities, and use these to set neural and synaptic parameter values, which may be less intuitive.

## 6 Discussion

In this paper, we presented analytical methods for setting parameters in dynamical neural networks that can add, subtract, multiply, divide, differentiate, and integrate incoming signals. Such operations are at the core of control, and these techniques enable control networks to be assembled rapidly and tuned directly. This work primarily identifies constraint equations, not unique values, that govern how parameters should be tuned. Thus, many different networks may perform the same function with different parameter values, as observed in real neural circuits (Prinz et al., 2004). Since these results are analytical, not based on machine learning or optimization, there is no concern about these networks over- or under-fitting training data, and their behavior is provable. These techniques build on our previous analysis of synthetic nervous systems (Szczecinski et al., 2017b) and have been validated through several studies with our robot, MantisBot (Szczecinski and Quinn, 2017; Szczecinski et al., 2017a).

All of the results from this paper make it easier to tune neuromechanical models of animals, as well. Many such models have been created to study the principles underlying insect (Daun-Gruhn and Tóth, 2010; Szczecinski et al., 2014) and mammalian (Hunt et al., 2015a; Markin et al., 2016) locomotion alike. Oftentimes, parameters of these models are tuned by hand to obtain the intended motion, which is a painstaking, slow, and imprecise process. The analysis in this paper can make neuromechanical models come together more quickly, and have more predictable behavior, leading to more thorough scientific investigations. More precise tuning methods enable more thorough validation or invalidation of hypotheses. Faster tuning methods enable more rapid validation or invalidation of hypotheses. For example, these methods could be used to improve the coordination our previous cockroach model (Szczecinski et al., 2014). In the model, curve walking of varying radii was achieved by modulating muscle activations with broad descending commands. However, the coordination, reliability, and repeatability of such motion could be improved with the methods of this paper, enabling us to improve or reject the model.

### 6.1 Simplifications

Some of the calculations in this paper are based on approximations, which lead to inaccuracies in the calculations of the subnetworks. One example is that the subtraction network does not produce linear output. This non-linearity occurs because the reversal potentials of synapses are rarely much lower than the resting potentials of neurons, requiring large values of *g*_*s*,2_ to build a subtractor where *k_syn_* = 1. A large *g*_*s*,2_ value increases *U*_*pre*,2_’s effect on the denominator of $U_{\mathrm{post}}^{*}$’s response, causing the synaptic input to reduce *U_post_*’s sensitivity to inputs. This is particularly noticeable in the differentiator’s response (Figure 4), especially as *k_syn_* increases.

Another example of a simplification we made is that our calculation of *k_i_* only used the particular solution of the system. This means that a transient response also exists, which we did not compute. In addition, *k_i_* is a function of *U*_1_ and *U*_2_. This means that *k_i_* is not a constant for this network. However, the impact of *U*_1_ and *U*_2_ on *k_i_* can be minimized by minimizing *g_s_* and maximizing *R*, as we showed in Sec. 4.2.

However, the developed networks are not intended to act as perfect analogs to their mathematical counterparts. These networks are intended to act as representations of real neural circuits, which likely do not act as perfect adders, multipliers, differentiators, etc. Dynamic and transient effects are a real part of biological control systems, and effective neural controllers have developed around these idiosyncrasies and have likely evolved to even exploit many of these aspects. In spite of these issues, the methods in this paper are valuable. Our recent robotics work (Szczecinski and Quinn, 2017; Szczecinski et al., 2017a), as well as related work in progress, is proof of the effectiveness of this approach.

### 6.2 Why Put Neurons in the Way?

The methods in this paper enable the direct construction of networks that perform arithmetic and dynamic calculations. Why bother building neural networks just to recreate mathematical operators? We believe there are several reasons to take this approach. From a neurobiology perspective, the constraints that we have identified may help explain why certain structures are common in the nervous system (David Friel, personal correspondence). For instance, mutually inhibitory parallel pathways are common in the thoracic control of insect locomotion (Büschges and Wolf, 1995), which may function as subtraction networks in negative feedback loops. As another example, networks in the retina of the rabbit are selectively sensitive to motion in one direction or the other (Barlow and Levick, 1965). Such a network could be constructed by using adjacent cells in the retina as inputs to differentiator networks. This would be consistent with both the function of direction-sensitivity, as well as the laterally inhibitive structure. Even though such consistency does not guarantee that the animal’s nervous system functions precisely this way, the design methods in this paper may aid in understanding the function of neural networks found in animals.

Additionally, the constraints that we identified may be used to constrain parameter values in large brain models. Rather than using global search techniques to understand the dynamics of a large pool of neurons, we believe it may be faster to begin with a number of functional subnetworks, and then use local search techniques to tune the connections between them. In this way, the designer is certain that parts of the network perform specific, useful computations, rather than naively optimizing a large network (Haferlach et al., 2007; Agmon and Beer, 2013; Izquierdo and Beer, 2013). The end result is something like a genetic program, but in a neuroscience context.

## Author Contributions

NS led research on functional subnetworks and led the preparation of the manuscript. AH aided in the preparation of the manuscript. RQ provided critical oversight of the research and aided in the preparation of the manuscript.

## Conflict of Interest Statement

The authors declare that the research was conducted in the absence of any commercial or financial relationships that could be construed as a potential conflict of interest.

## Appendix

### A Derivation of Integrator Eigenvalues and Eigenvectors

We find the eigenvalues λ_1_ and λ_2_ and the associated eigenvectors *X*_1_ and *X*_2_ of the Jacobian matrix by the eigenvalue problem,

(A1) $$\det\left(J-\lambda_{i}\cdot I\right)=0$$

(A2) $$J\cdot X_{i}=\lambda_{i}\cdot X_{i},$$

where *i* is the index of the eigenvalue (1 or 2), $I\in R^{2\times 2}$ is an identity matrix, and *J* is the square matrix from equation (64). Solving for λ,

(A3) $$\lambda_{1}=0,\;\lambda_{2}=\frac{a+b}{C_{m}}>0.$$

Because *J* is on the same side of the equation as $\dot{\vec{x}}$ (see equation (65)), λ_2_ > 0 indicates a stable system (e.g., as the stiffness matrix of a physical system). $\lambda_{2}>0\forall \vec{x}$, because *a* > 0 and *b* > 0. The definition of *a* in equation (62) shows that *a* > 0 because *g_s_* > 0 (it is a physical quantity) and *U*_1_/*R* ∈ [0, 1]. The same reasoning applies to *b*.

We use the eigenvalues to find their associated eigenvectors,

(A4) $$X_{1}=\left[\begin{array}{c} 1\\ -b∕a \end{array}\right]\;\;.$$

Normalizing *X*_1_ to 1,

(A5) $$X_{1}=\left[\begin{array}{c} \frac{1}{\sqrt{1^{2}+{\left(-b∕a\right)}^{2}}}\\ \frac{-b∕a}{\sqrt{1^{2}+{\left(-b∕a\right)}^{2}}} \end{array}\right]=\left[\begin{array}{c} 1∕d\\ -b∕\left(\mathrm{ad}\right) \end{array}\right]\;\;,\;d=\sqrt{1^{2}+{\left(-b∕a\right)}^{2}}.$$

Next, we calculate

(A6) $$X_{2}=\left[\begin{array}{c} 1\\ 1 \end{array}\right]\;\;.$$

Normalizing *X*_2_ to 1,

(A7) $$X_{2}=\left[\begin{array}{c} 1∕\sqrt{2}\\ 1∕\sqrt{2} \end{array}\right]\;\;.$$

We now know the transformation matrix between the natural coordinates, $\vec{x}={\left[U_{1},U_{2}\right]}^{T}$, and the generalized coordinates, $\vec{q}={\left[q_{1},q_{2}\right]}^{T}$:

(A8) $$\vec{x}=X\cdot \vec{q},\;X=\left[X_{1},X_{2}\right]=\left[\begin{array}{cc} 1∕d & 1∕\sqrt{2}\\ -b∕\left(\mathrm{ad}\right) & 1∕\sqrt{2} \end{array}\right]\;\;.$$

We will also make use of *X*^−1^ when transforming between natural and generalized coordinates. We can analytically invert *X* from equation (A8),

(A9) $$X^{-1}=\frac{a\cdot d\cdot \sqrt{2}}{a+b}\cdot \left[\begin{array}{cc} 1∕\sqrt{2} & -1∕\sqrt{2}\\ b∕\left(\mathrm{ad}\right) & 1∕d \end{array}\right]=\left[\begin{array}{cc} \frac{a\cdot d}{a+b} & \frac{-a\cdot d}{a+b}\\ \frac{b\cdot \sqrt{2}}{a+b} & \frac{a\cdot \sqrt{2}}{a+b}. \end{array}\right]$$
